# Policing Fish at Boston's Museum of Science: Studying Audiovisual Interaction in the Wild

**DOI:** 10.1177/2041669515599332

**Published:** 2015-08-21

**Authors:** Hannah Goldberg, Yile Sun, Timothy J. Hickey, Barbara Shinn-Cunningham, Robert Sekuler

**Affiliations:** CompNet, Boston University, USA; Psychology, Brandeis University, Waltham, USA; Computer Science, Brandeis University, Waltham, USA; Biomedical Engineering, Boston University, USA; Volen Center for Complex Systems, Brandeis University, Waltham, USA

**Keywords:** audiovisual interaction, temporal structure, multisensory, video games

## Abstract

Boston's Museum of Science supports researchers whose projects advance science and provide educational opportunities to the Museum's visitors. For our project, 60 visitors to the Museum played “Fish Police!!,” a video game that examines audiovisual integration, including the ability to ignore irrelevant sensory information. Players, who ranged in age from 6 to 82 years, made speeded responses to computer-generated fish that swam rapidly across a tablet display. Responses were to be based solely on the rate (6 or 8 Hz) at which a fish's size modulated, sinusoidally growing and shrinking. Accompanying each fish was a task-irrelevant broadband sound, amplitude modulated at either 6 or 8 Hz. The rates of visual and auditory modulation were either Congruent (both 6 Hz or 8 Hz) or Incongruent (6 and 8 or 8 and 6 Hz). Despite being instructed to ignore the sound, players of all ages responded more accurately and faster when a fish's auditory and visual signatures were Congruent. In a controlled laboratory setting, a related task produced comparable results, demonstrating the robustness of the audiovisual interaction reported here. Some suggestions are made for conducting research in public settings.

## Introduction

For more than a century, researchers have occasionally collected psychophysical data outside the laboratory, in public or quasi-public settings. Notwithstanding the logistical challenges they present, such environments can be attractive, especially for research focused on developmental trends and individual differences. Perhaps the best-known example of research done in a public setting is Galton's (1895) study of 9,000 visitors to the London International Health Exhibition. After paying a small fee for the privilege, each visitor's visual acuity, hearing, reaction time, and other functions were measured.

The Living Laboratory, located in Boston's Museum of Science, provides a unique research environment. The Museum invites scientists from local universities and hospitals to use the Laboratory as a venue for engaging visitors in ongoing research that also offers visitors a valuable educational experience. The Laboratory makes it possible to collect useful data from large and diverse samples of subjects across a wide age range, from school-aged children through senior citizens. For some time, we have been interested in audiovisual integration, particularly the ways in which a correlation between signals' temporal structures promotes their integration (Parise, Spence, & Ernst, 2012). So we took advantage of the Museum environment in order to examine age-related changes in audiovisual integration, and also to test the robustness of observations made previously, in well-controlled laboratory settings (Benussen et al., 2014; Sun, Shinn-Cunningham, Somers, & Sekuler, 2014).

Psychophysical experiments are often time consuming and usually require many repetitions of the same measurement. As a result, test subjects must be available, attentive, and motivated for one or more lengthy testing sessions. Particularly when researchers want to study a wide age range of participants, the repetitive, sometimes monotonous aspects of an experiment can cause participants to withdraw before the needed data have been collected. Withdrawal before testing is complete can render data collected up to that point unusable. To minimize such risks, we embedded our experiment within a simple video game, which was designed to engage and amuse participants while also generating data on interactions between what subjects saw and what they heard (e.g., Abramov et al., 1984; Miranda & Palmer, 2014).

**Figure 1.**
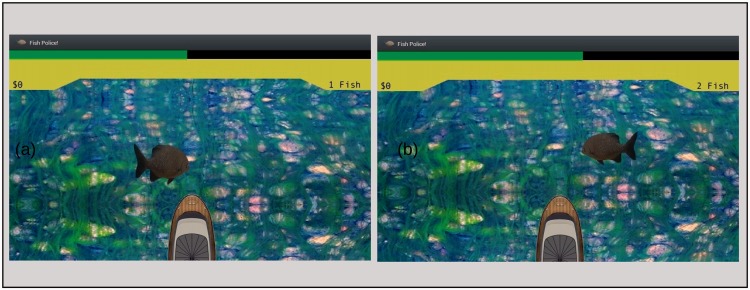
A pair of screen captures from the tablet display. (a) Fish appeared at the screen's left side. (b) Fish appeared at the screen's right side. The green countdown bar at the top of screen shows that more time is left before the response deadline in (b) than in (a). This difference corresponds to the fact that fish in (a) has moved further from its starting location (at the edge of screen) than has the fish in b. The size difference between the fish results from the fact that the screen shot shows the two fish at different points in their size oscillation cycles.

In our video game, “Fish Police!!,” players watched as computer-generated fish appeared one at a time and swam rapidly across a virtual river (see Figure 1). As it swam, each fish oscillated sinusoidally in size, at either 6 or 8 Hz. To make the task harder, each fish's path was perturbed by a series of small random vertical displacements. Accompanying each fish was a broadband sound that was amplitude modulated at either 6 or 8 Hz. Subjects were instructed to classify each fish as rapidly as possible using only what they saw, judging whether a fish oscillated at the slower (6 Hz) or faster rate (8 Hz). While making these judgments, subjects were to ignore the concurrent amplitude modulated sound. If, despite these instructions, the sound affected subjects' responses, we expected that categorizations to be more accurate and faster when visual and auditory signals were Congruent, that is when they shared the same rate, rather than Incongruent, when auditory and visual signals were mismatched in rate.

Once a fish appeared and began its journey across the river, a subject had just 2 seconds to respond before the fish would disappear from view. Then 3 seconds later, a new fish appeared and began its journey across the river. This schedule spawned about 12 fish per minute, which made the game challenging and seemed to promote sustained attention.

In addition to a task design that would be accessible to subjects of all ages, this unique setting also required special considerations in terms of set-up. In laboratory environments, equipment needed for research can be set up and left unmolested for repeated use over an extended period. In public settings, that is usually impossible to do. The Living Laboratory's space is time-shared among multiple research projects, each of whom can use the space only a few hours each week. At the end of each day's assigned time, researchers must pack up and remove all the equipment and materials that had been used. To accommodate this requirement, our experiment's game was implemented in Python on an inexpensive touchscreen tablet computer (Samsung Note 10.1) running the Android mobile operating system.

To boost players' enjoyment, Fish Police!! incorporated several features common to video games (Hawkins, Rae, Nesbitt, & Brown, 2013; Miranda & Palmer, 2014). For example, each correct response was followed immediately by a pleasant, rewarding sound (clinking of coins), and each incorrect response brought an unpleasant sound (a short buzz). Additionally, a running total of correct responses, represented by a collection of coins, was displayed at the top of the computer tablet screen (see Figure 1). The length of a green progress bar near the display's top indicated the time remaining before the response deadline. Thanks to these features and the task's inherent challenge, Fish Police!! proved sufficiently engaging that of the Museum visitors who began play, only ∼10% quit before completing the game. Of these, approximately half were either ushered away by parents or guardians or were interrupted by some other unavoidable event. Moreover, about 25% of the players asked if they could play a second time, a request that we had to decline because there was almost always a line of people waiting their turn to play.

As potential players of Fish Police!! would be unused to the attentional demands of psychophysical experiments, we decided to embed subjects' instructions in a narrative that would be engaging, easily understood, and easily remembered. After being shown the computer tablet on which the game would be played, subjects were told:

You are going to be the police officer in charge of a river. One at a time, from either side of the tablet, a fish will appear. They are very nervous, though, because they don't want to be caught by you! The bad fish will be wiggling fast because they're scared, that's when you tilt the tablet towards you to catch them. If the fish is wiggling more slowly, then it's a good fish because it isn't as nervous, so you can tilt the tablet away from you to let it go. Remember though, since they've been swimming for a while, they're a little bored so they hum --you'll hear this through the headphones I'll be putting on you. Try as best you can to JUST focus on their wiggling to tell if they're a good or bad fish.

Each participant played the game while holding the tablet with one hand on each side of the screen. This made it possible to implement a pair of responses, tilting the tablet either toward or away from the player that had an easily remembered correspondence to the judgment that each direction signaled.

At the Living Laboratory's entrance, a video monitor advertized the game by displaying an image from Fish Police!!'s splash screen. Before commencing play, a player's basic demographic information, initials and age, was entered via the tablet's touchscreen. This information and all data generated during game play were de-identified and uploaded wirelessly by the tablet in real time to a secure server offsite. The tablet's built-in accelerometer sampled the tablet's angle of tilt at 60 Hz. The Python script controlling the video game defined a response as a rotation that was 17 ° or more relative to the tablet's orientation at the moment when a fish first appeared on the screen. This rotation threshold guarded against that possibility that spurious, unintended movements of the tablet would be mistaken for genuine responses to the fish. Once the Python script detected that the response threshold had been exceeded, it triggered the appropriate feedback for players, either the sound of coins clinking or a buzzing sound, and then, 3 seconds later, spawned the next fish. All accelerometer readings and responses were inserted into a data stream sent wirelessly from the testing area to the offsite server.

Before beginning the game, a player was shown how to hold the tablet, with one hand on each side, and how the tablet was to be tilted in order to signal a response. Then, each player was given three practice trials with Congruent and Incongruent “good” and “bad” fish. Also included in these practice trials were some in which the fish was presented with no accompanying sound. These no-sound trials were meant to reinforce the point that the player was to judge a fish only on the basis of visual attributes. The practice trials provided an opportunity to ensure that a player understood the task. These trials were followed by a set of 10 practice trials in which conditions were randomized. Subjects scoring six or more correct on these randomized trials were allowed to move on to the game proper; 12 subjects who failed to reach that threshold received an additional set of 10 practice trials. Most of these 12 were among the younger subjects, who may have been particularly distracted by the presence of schoolmates and friends. After a player finished, the player's proportion of correct responses and mean response time, along with the initials and results of the previous 10 players, were displayed on a large computer monitor visible to the player and to any onlookers. Video 1 affords a player's eye perspective on the game.

## Experimental Design and Results

**Figure 2.**
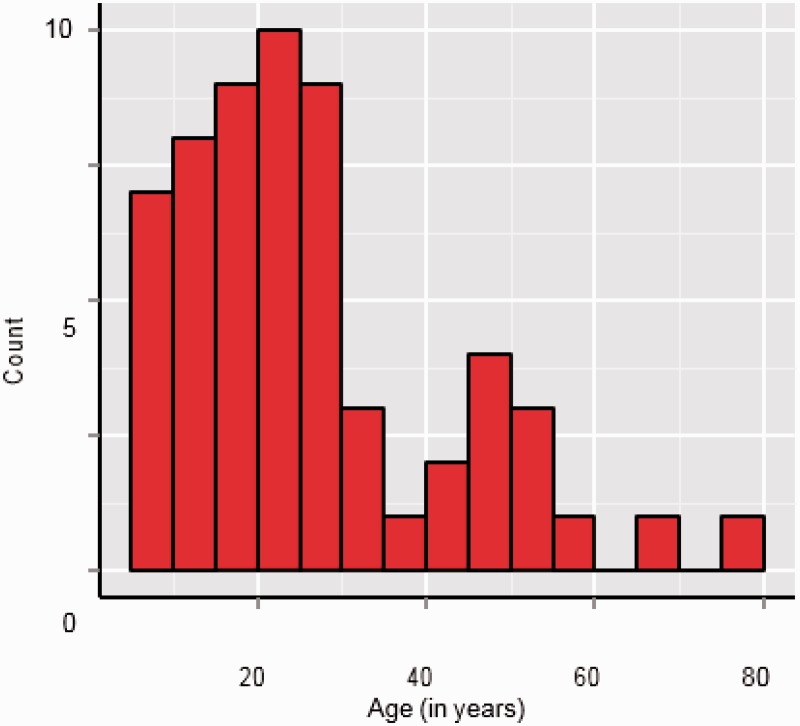
Age distribution of participants who completed the entire 5-minute game. Each bin spans 10 years.

During the approximately 5 minutes of play, each player had the opportunity to judge and respond to 60 fish. For half the fish, the frequency of its size oscillation and the frequency of the concurrent amplitude modulation were Congruent, that is, both were the same, either 6 Hz or 8 Hz; for the other half of the fish, size and sound amplitude modulation were Incongruent, that is, they were at different frequencies. Specifically, the fish sinusoidally oscillated in size, either at 6 Hz or 8 Hz, while the concurrent sound amplitude modulated at the other frequency, that is, 8 Hz or 6 Hz. For indices of audiovisual integration, we contrasted players' performance with Congruent fish to their performance with Incongruent fish, using as metrics the proportion of categorizations that were correct, and the latency of response on correct trials; 60 test participants completed the entire 5-minute game; their ages ranged from 82 years down to 6 years, the youngest age we had permission to test. Figure 2 shows the age distribution of the players.

The 60 fish each player saw and heard were uniformly distributed across four categories defined by the two species of fish (good fish—6 Hz oscillation in size and bad fish—8 Hz oscillation in size), crossed with two types of congruence (Congruent audiovisual signals and Incongruent audiovisual signals). For each subject, this 2 × 2 design produced just 15 trials per cell, too few samples for stable estimates of the dependent measures. For example, with only 15 samples and binomial variability, a change of just a single response would produce a swing of more than 13%. To reduce the impact of having so few samples per condition, while maintaining the focus on audiovisual interaction, we aggregated responses into just two categories: All fish whose audiovisual signals were Congruent and all fish whose audiovisual signals were Incongruent. In so doing, we ignored the frequency at which any particular fish varied in size.

**Figure 3.**
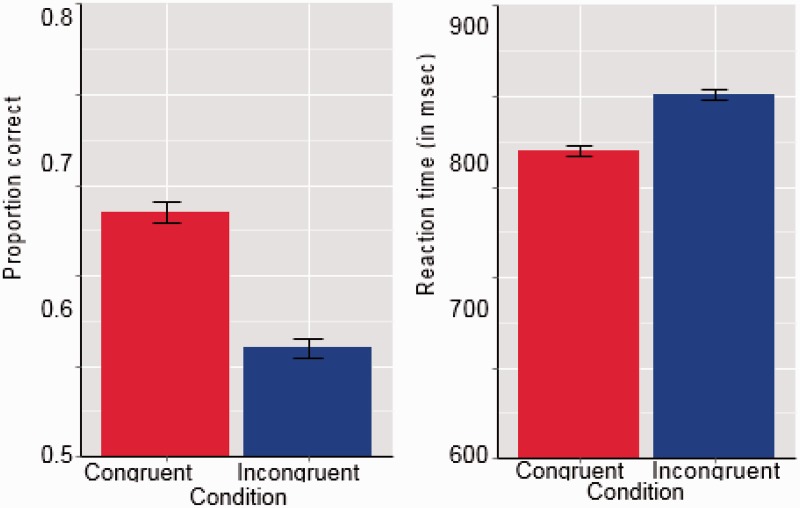
Left: Mean proportion correct responses for Congruent and Incongruent Fish. Right: Mean reaction time for Congruent and Incongruent Fish. Error bars are within-subject standard errors of the mean.

Data analysis began by comparing the accuracy with which players responded to Congruent and Incongruent fish. Audiovisual Congruent fish were correctly categorized more often than were Incongruent fish. The mean difference between the two proportions correct was 0.15 (95% CI [0.11, 0.20]) (Figure 3). Differences between proportions of correct responses made to Congruent and Incongruent fish were entered into a one-sample *t* test, producing *t* = 6.89, which corresponds to *p* = 4.128e-09 (with *df* = 59) (Figure 3).

We next focused on response times from trials on which correct responses were made. Mental chronometry (Posner, 2005) treats a response time as a composite of separable components. Commonly, such components include movement time (MT), the time required to execute a motor response, and reaction time, the time needed to perceive and process the stimulus and to select the response. As Doucet and Stelmack (1999) noted, reaction time is influenced by the information processing demands of the subject's task, while MT may not be. Fish Police!!'s accelerometer records allowed us to estimate MT. The accelerometer's tilt reading was reset to zero when a fish first appeared, providing a baseline against which all subsequent tilts were evaluated. We examined successive 60 Hz samples generated by the tablet's accelerometer in order to find the time sample at which the tablet was first tilted 17 ° either toward or away from the player. As explained earlier, this time sample defined the trial's response time. Later, an algorithm made an offline search of the stored accelerometer samples from each trial in order to find the trial's first sample that satisfied our criterion: The sample represented the tilt of the tablet in the same ‘direction’ as the trial's ultimate 17 ° tilt, and the sample was not followed by any reversal in direction of tilt. The time associated with the sample that satisfied both these criteria was used to define the trial's reaction time (Table 1). The trial's MT was defined by the difference between the trial's reaction time and its response time.

The mean MT for Congruent fish was 175.58 ms; the corresponding mean for Incongruent fish was 162.88 ms. The difference between these two means was not statistically significant, *t* = 0.93, *p* > 0.35 (*df* = 59), (95% CI for the difference, [−14.49 and 39.91]). Note that the 60 Hz sampling rate of the accelerometer introduced some irreducible uncertainty into our mental chronometric estimates. Moreover, one subject continuously jiggled the handheld tablet, making it impossible to extract reaction time and MT from his/her records; that subject's data were excluded from further analysis.

Each trial's MT was subtracted from its response time, yielding the reaction time for that trial. Thereafter, all chronometric analyses were done on values of this derived reaction time variable. Players' reaction times were significantly shorter for Congruent fish than for Incongruent ones. The mean difference between the two sets of reaction times was 59.21 ms (95% CI [39.50, 78.95]). A *t* test on the differences between the two sets of reaction times produced *t* = 6.00 (*df* = 59), for *p* = 1.277e-07. So both dependent measures, time and accuracy, produced reliable differences between players' processing of Congruent and Incongruent fish (Figure 3).

**Figure 4.**
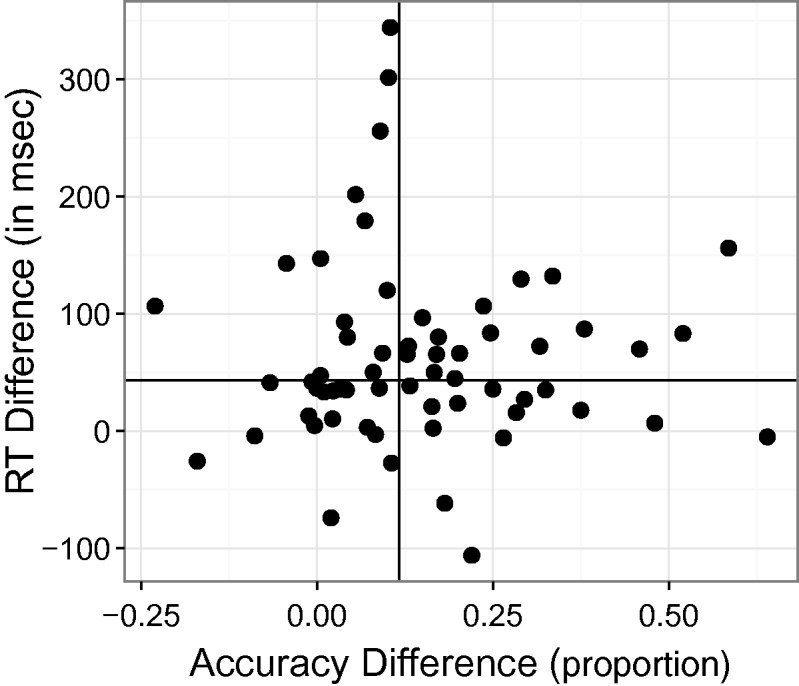
Speed-accuracy relationship. Effect of audiovisual congruence defined by differences in reaction time plotted against the effect defined by differences in accuracy of response. Data points are for individual players. The vertical line separates the half of subjects whose accuracy-defined congruency effects were largest and the half of subjects whose effects were lowest; the horizontal line separates the half of subjects whose reaction time-defined congruency effects were largest and the half of subjects whose effects were smallest. If there were zero correlation between the two, data points would tend to be equally distributed among the graph's four quadrants.

We were interested in the possibility that subjects might have been trading accuracy for speed of response (Beilock, Bertenthal, Hoerger, & Carr, 2008; Heitz, 2014; Wickelgren, 1977). Viewing each fish for a longer time could have allowed additional visual information to accumulate, thereby increasing the proportion of correct responses (Mazurek, Roitman, Ditterich, & Shadlen, 2003; Noppeney, Ostwald, & Werner, 2010). More recently, Teichert, Ferrera, and Grinband (2014) showed that subjects strategically increase response accuracy by delaying the onset of their decisions. Would players whose reaction times were long tend to produce a higher proportion of correct responses? To evaluate this potential connection between players' speed and accuracy, we examined the correlation between the two measures. In Figure 4, the effect of audiovisual congruence as represented by reaction time is plotted against the effect of audiovisual congruence as represented by accuracy. These two measures of the congruency effect were not significantly correlated, *r* = −0.006; the (95% CI included zero [−0.255, 0.244]). So, whatever factors contributed to differences in the way that players were affected by audiovisual congruence, these factors did not include adoption of some consistent speed-accuracy strategy.

**Figure 5.**
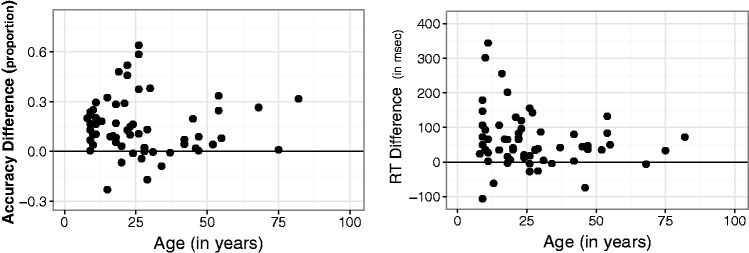
The effect of congruence between visual and auditory signals as a function of player age. Left: Effect of congruence expressed as the difference between percent correct judgments when auditory and visual frequencies were Congruent and when they were Incongruent. Right: The effect of congruence expressed as the difference in reaction times for Congruent and Incongruent fish.

Previous studies suggested that in humans, audiovisual integration emerges late in the first year of life (Neil, Chee-Ruiter, Scheier, Lewkowicz, & Shimojo, 2006), but continues to be fine-tuned until somewhat later. Moreover, at least one study (Roudaia, Sekuler, Bennett, & Sekuler, 2013) showed weakened audiovisual interaction in older adults. All of these studies used audiovisual stimuli and tasks that were quite different from those embodied in our game. Although we were limited to testing subjects 6 years of age and older, we thought it would be worthwhile to evaluate how audiovisual interaction in Fish Police!! varied with age. Figures 4 and 5 show the results of this inquiry for accuracy and reaction time measures, respectively. The slopes and confidence intervals from linear regressions showed that neither congruence effect was statistically significant: for reaction time, *p* = 0.13, and for accuracy, *p* = 0.82. The 95% confidence intervals around the slope of regression against players' age each included zero, [−0.0029, 0.0023] and [−2.12, 0.28], for accuracy and response time measures of congruence, respectively.

## Discussion

Results with each dependent measure were consistent with those from a comparable task studied in a controlled, laboratory setting (Sun et al., 2014). In addition to differences in the level of distractions, the experiments comprising the laboratory study differed in multiple ways from the conditions in the Museum of Science. For example, they differed in size of the display (33 ° vs. ∼14–15 ° visual angle wide), response modes (button press vs. tablet tilt), number of trials each subject experienced (∼300 vs. 60), interfish intervals (∼2 vs. 3 seconds), and number of subjects per experiment (10 vs. 60). Table 2 shows that despite these differences, the main outcomes of the present study are not dramatically different from what was seen in the three laboratory experiments. The absence of strong differences among venues and conditions points to the robustness of audiovisual integration that arises from temporally correlated auditory and visual signals.

**Table 1.** Response and Reaction Times.

	Congruent	Incongruent
Response times		
Mean	1115.26	1164.91
SEM	17.95	22.38
Reaction times		
Mean	939.67	1002.03
SEM	20.45	20.11

**Table 2.** Accuracy and Reaction Time Results From the Present Study and From Three Laboratory Experiments.

Source	Difference in *pr* (correct)	Difference in reaction time (in ms)
Museum	0.15	59
Experiment 1	0.15	88
Experiment 2	0.11	100
Experiment 3	0.20	74

*Note*. Each entry is the absolute value of the mean difference in responses to Congruent and Incongruent fish.

Moreover, results from the Museum and from the laboratory show that the effect of temporal correlation of auditory and visual signals is strong enough to survive even with no corresponding spatial correlation between visual and auditory information. That is, the spatial information provided by vision (the fish's progress across the virtual stream) was not matched by a comparable change in auditory spatial information (such as a change in interaural time difference).

As explained above, results from the two dependent measures (Figure 4) suggest that differences among players' accuracy could not be explained simply by differences in their strategies for balancing the competing demands for speed and accuracy. It seems likely that the time constraints imposed by the game design (allowing just 2 seconds to respond) created high pressure that might have a particular effect on players tested at the Museum of Science. Each player had just a few minutes total experience with the game. As a result, it may be that the time pressure had a greater impact on them than it would have had on more experienced players (Beilock et al., 2008). Although we cannot be certain, it is plausible that instructions that mentioned the cost of a missed response and called attention to the time varying green progress bar near the top of the display encouraged players to prioritize speed of response. That speculation is consistent with the observation that very few responses, only 28 out of 3,600 (.009), failed to beat the deadline.

In our video game, whenever a fish's auditory and visual attributes were not congruent, the rate at which the fish's sound was amplitude modulated not only did not match the rate of its size oscillation but also actually matched the visual modulation rate of fish from the competing type, either “good” or “bad” species. This particular form of mismatch between auditory and visual signals was intended to maximize stimulus-response interference (Fitts & Deininger, 1954), which was expected to maximize observed differences between responses to Congruent and Incongruent fish.

Planning this project made us mindful of the many differences between a public or quasi-public research environment and the well-controlled dedicated research laboratories in which we have been studying various aspects of audiovisual interactions (Benussen et al., 2014; Sun et al., 2014). Anticipating that other researchers might want to carry out a project in a setting like the one we found at the Museum, we offer some suggestions for optimizing such opportunities.

Of course, visual distractions and background noise are unavoidable in public spaces. In the Living Laboratory, visual distractions included the Museum's nearby scientific attractions, the stream of curious passers-by, as well as players' friends, relatives and, for school age players, their classmates, and teachers. To minimize these and other potential visual distractions, we took the simple step of seating participants so that they faced a blank wall, with backs turned toward onlookers. Appreciable noise was generated by nearby interactive exhibits and by the chatter of excited visitors to Museum, particularly younger visitors. These sounds were consistently ∼76 to 80 dB SPL. To control the impact of audio distractions, participants wore Bose QuietComfort 15 Acoustic Noise Canceling Headphones while playing the game. These provided ∼30 dB attenuation. This reduction in background sound helped subjects focus on the task, with reduced influence from the occasional heckling or encouragement of family or friends, or by the general din of the Museum.

As mentioned earlier, a publicly viewable video monitor displayed each player's initials, score, along with the scores of the previous 10 players. This leader board seemed to motivate players, particularly when a pair of museum-goers decided that they would be competing against one another. The leader board was helpful also to players who did not do well, as it showed them that there was considerable variation among players. In this regard, the leader board was particularly helpful to parents of children whose play was good, but not perfect. A less than perfect score made some players' parent ask what was “wrong” with their child. Seeing the scores of the preceding 10 players helped to provide a reassuring answer to that question. Finally, the leader board encouraged some players to ask a scientifically important, but difficult question, namely, why success varied as it did from one player to another. For that question, we have yet to develop a satisfactory answer.

Even if some planned project does not entail auditory stimuli, researchers might still consider some technique to attenuate extraneous sounds. The noise canceling headphones worn while subjects played Fish Police!! were particularly useful, as they served two purposes. First, the headphones reduced the saliency of the extraneous sounds that were plentiful in the Museum setting; second, the headphones signaled to onlookers, particularly friends or family members, that the headphone's wearer was engaged in an important task and therefore should not be disturbed.

In settings like the Museum of Science, many visitors are eager to take part in an activity that simultaneously is enjoyable and advances science. An experiment should be engaging and provide a vehicle for subjects to become interested in science and learn about real world applications of research. Embedding an experiment in a game is one effective way to achieve these goals. As researchers, we were gratified that so many of the Museum visitors who played Fish Police!! were interested in how their data would be used and how their results would impact science. For their participation and contribution, we are most appreciative.
